# Synchronous bilateral breast cancer: A case report of heterogeneous estrogen receptor status

**DOI:** 10.1016/j.ijscr.2018.10.016

**Published:** 2018-10-24

**Authors:** Sunny Dhadlie, Joseph Whitfield, Rasika Hendahewa

**Affiliations:** a Caboolture Hospital, 120 McKean Street, 4510, Queenland, Australia; b QML Pathology, 11 Riverview Place, Murrarie, 4172, Queensland, Australia

**Keywords:** Synchronous, Bilateral breast cancer, Hormone receptors, Oestrogen receptor

## Abstract

**Introduction:**

Tumour heterogeneity is important in the management of breast cancer. Hormone receptors are established biomarkers for treatment and prognosis of patients with breast cancer.

There are three immunohistochemical biomarkers: estrogen receptor (ER), progesterone receptor (PR) and human epidermal growth factor 2 (HER2).

We explore whether heterogeneity in hormone receptor status in synchronous bilateral breast alters therapeutic management.

**Case presentation:**

This case details a 54 year old woman who was referred to our clinic by her general practitioner for investigation of bilateral breast pain that she had for 6 months.

On clinical examination pathological nodes were palpated in bilateral axilla. There was left sided nipple inversion with a palpable mass in the upper outer quadrant of approximately 3 cm diameter.

On examination of the right breast there was skin tethering of the nipple and 3 masses were palpated, the largest being in the upper inner quadrant at 5 cm diameter.

Ultrasound and mammography of bilateral breasts demonstrated advanced bilateral breast cancer with axillary node metastases.

Core biopsies demonstrated invasive carcinoma. The right breast lesion was ER negative whilst the left breast lesion was ER positive.

**Discussion:**

In patients with synchronous bilateral breast cancer ER discordance in patients have been associated with higher mortality than ER concordant positive patients and lower mortality than ER concordant negative patients within the first 5 years of surveillance [1].

**Conclusion:**

Heterogeneity in hormone receptor status alters the therapeutic management of patients with synchronous bilateral breast cancer. Both hormone therapy and chemotherapy should be considered in these patients.

It is of utmost importance to evaluate the tumor receptor status in cases of synchronous bilateral breast tumour and to assess for change in relation to tumour progression or treatment. Further study in the status change of receptors could open up new treatment modalities.

## Highlights

• Hormone receptors are established biomarkers for treatment and prognosis of patients with breast cancer.
• Receptor status change is dynamic and unstable throughout tumour progression and during advance stage disease.
• Four mechanisms of breast cancer heterogeneity have been described which includes differentiation of state of cell origin, cell plasticity, genetic evolution of cancer and tumour microenvironment.

## 1 Introduction

Tumour heterogeneity is important in the management of breast cancer. Hormone receptors are established biomarkers for treatment and prognosis of patients with breast cancer.

There are three immunohistochemical biomarkers: estrogen receptor (ER), progesterone receptor (PR) and human epidermal growth factor 2 (HER2).

We explore whether heterogeneity in hormone receptor status in synchronous bilateral breast alters therapeutic management.

This case has been reported in line with the SCARE criteria [1].

## 2 Case presentation

This case details a 54 year old woman who was referred to our clinic by her general practitioner for investigation of bilateral breast pain that she had for 6 months.

Her past medical history included hypertension for which she took a single anti-hypertensive. She had no familial history of breast or gynaecological malignancy. On clinical examination pathological nodes were palpated bilaterally in the axillae. There was left sided nipple inversion with a palpable mass in the upper outer quadrant of approximately 3 cm diameter.

On examination of the right breast there was skin tethering of the nipple and 3 masses were palpated, the largest being in the upper inner quadrant at 5 cm diameter.

Ultrasound and mammography of bilateral breasts demonstrated advanced bilateral breast cancer with axillary node metastases.

The right breast had a large lesion consistent with primary breast cancer at 12 o’clock measuring 44.3 mm in diameter. An additional 7 smaller lesions were distributed through the right breast consistent with satellite lesions. The largest pathological node in the right axilla measured 42 × 30 mm.

In the left breast at the 2 o’clock position there was a lesion consistent with breast carcinoma measuring 31.2 mm. The largest node in the left axilla measured 13.1 × 10.2 mm.

**Fig. 1:**
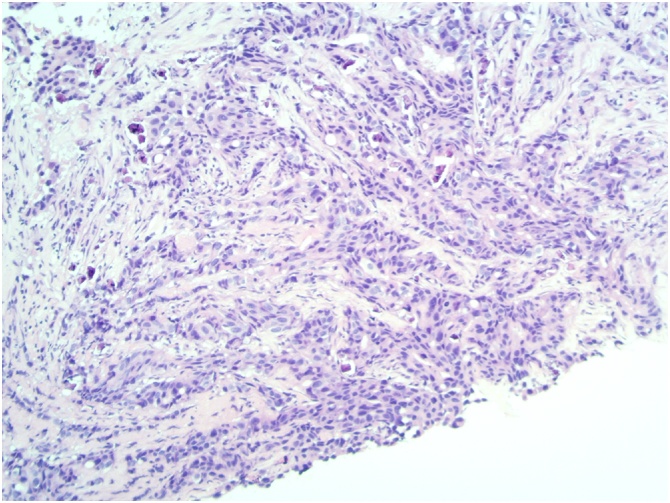
Left core biopsy.

**Fig. 2:**
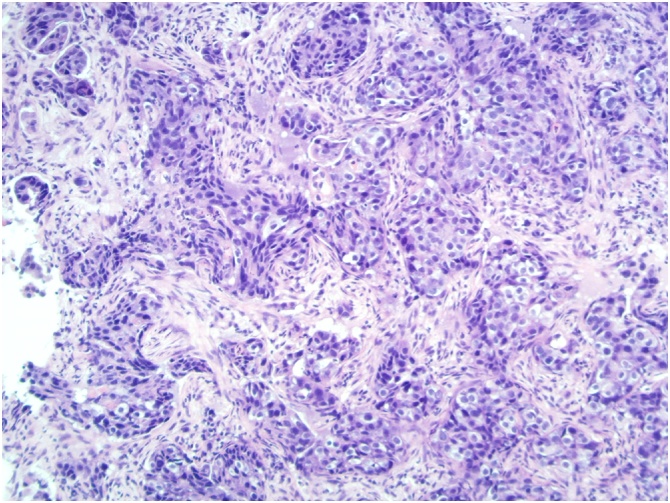
Right core biopsy.

There was skin thickening of both breasts consistent with oedema, the right side was most pronounced. Core biopsies from lesion on right breast at 12 o’clock and the left breast at 2 o’clock demonstrated invasive carcinoma. Fig. 1, Fig. 2: (H&E x10) Left/right core biopsy show invasive carcinoma, no special type

The hormone receptor status was identified with right breast lesion being ER negative whilst the left breast lesion was ER positive.

**Fig. 3:**
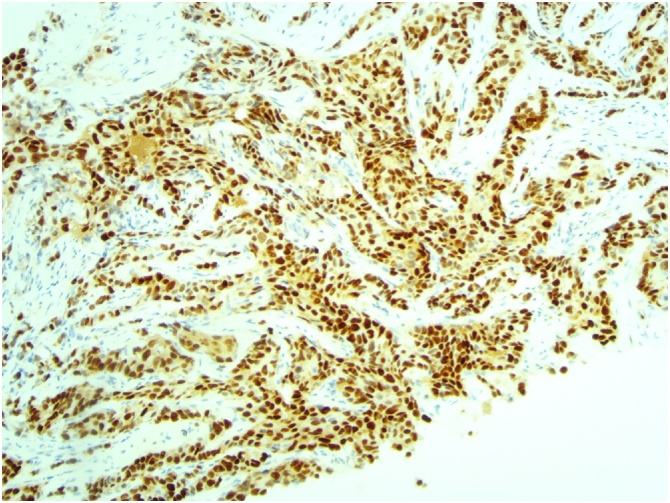
Left ER.

**Fig. 4:**
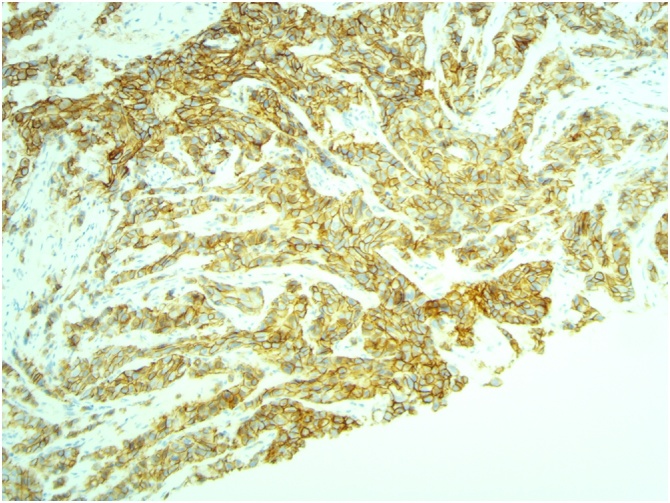
Left PR.

**Fig. 5:**
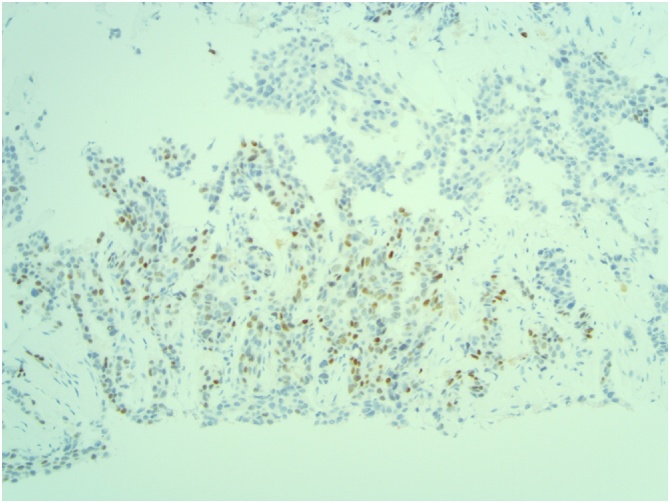
Left HER 2.

Fig. 3, Fig. 4, Fig. 5: (x10) Positive staining for ER and PR. HER2 Immunohistochemistry score 3 + .

**Fig. 6:**
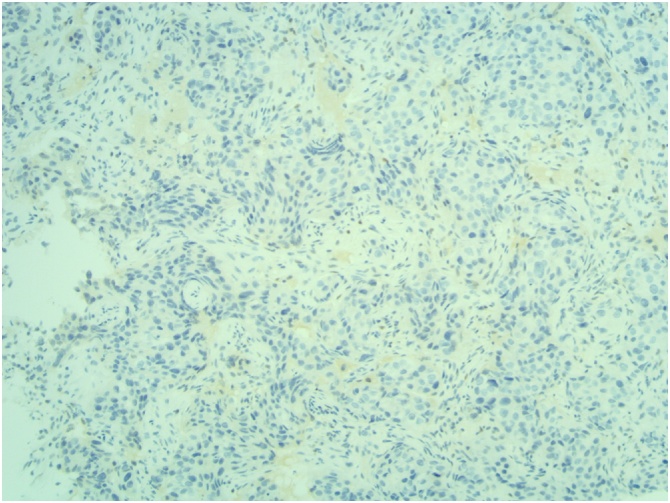
Right ER.

**Fig. 7:**
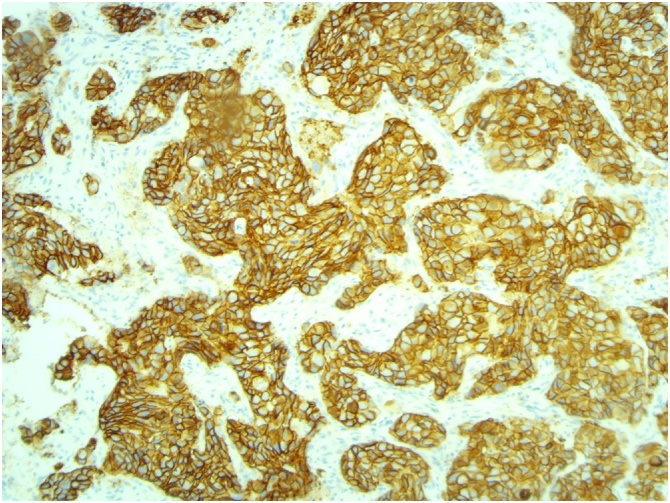
Right PR.

**Fig. 8:**
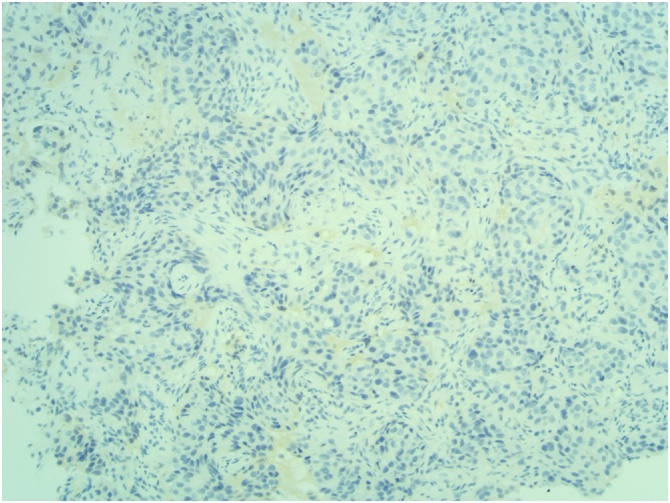
Right HER2.

Fig. 6, Fig. 7, Fig. 8: (x10) Negative staining for ER and PR. HER2 Immunohistochemistry score 3 + .

The patient was presented at an oncology multidisciplinary team meeting.

Subsequent staging scans were arranged which included a CT of head, neck, abdomen and pelvis, a nuclear medicine bone scan and a PET scan.

The CT scan showed no evidence of metastatic disease. The only finding was the bilateral breast malignancies with bilateral pathological axillary lymph nodes, with the right being most pronounced.

There was mild FDG avid ill defined ground glass pulmonary opacity in the left lower lobe of her lung, this was though to represent an inflammatory or infective process given its absence on the CT scan. A follow up CT scan was recommended to ensure resolution.

There was no evidence of bony metastases on the nuclear medicine bone scan.

The patient was referred to medical oncology to commence neo adjuvant chemotherapy and hormone therapy. Pending the response to the therapy the patient will be considered for bilateral mastectomy and axillary clearance.

## 3 Discussion

Tumour heterogeneity is one of the hallmarks of malignancy [2].

Intertumour heterogeneity is observed in breast carcinoma from different individuals. The presence of heterogenous cell populations within an individual tumour is referred to intratumour heterogeneity.

Intertumour heterogeneity is illustrated by clinical disease staging using physical examination and imaging findings. The TNM staging system by the American Joint Committee on Cancer/ Union for International Cancer Control incorporated the size of the tumour, the status of regional lymph nodes and presence of distant metastases [3].

Standard breast cancer treatment is based on clinical stage including histopathologic features and biomarker profile and tumour characteristics. Treatment is also affected by menopausal status, the patient’s age and general health. These factors have a significant impact on survival and account for most of the differences seen in clinical outcome among patients with breast ca [4,5].

Histopathologic classification of breast cancer is based on the morphologic heterogeneity of breast cancer. Invasive ductal carcinoma (IDC) not otherwise specified or of no special type (NOS) is the most common histologic type of invasive breast cancer (40–75%)^2^ In addition to IDC there are 21 special subtypes of breast cancer defined by the World Health Organisation (WHO) in 2012, of which invasive lobular carcinoma (ILC) is the most frequent (15%).^5^ The other special subtypes of breast carcinoma are much less common and differ greatly in relation to prognosis and response to adjuvant treatment [[6], [7], [8], [9]].

Papillary, mucinous and tubular carcinomas usually have excellent outcomes in comparison to IDC and ILC and are not always treated with chemotherapy. Metaplastic carcinoma and poorly differentiated IDC NOS have worse outcomes and systemic chemotherapy is used routinely [5].

Tumour heterogeneity is also highlighted by the grade of breast carcinoma. Grade is classified as low, intermediate or high in a 3-tier system. This is based on the evaluation of the percentage of tumour arranged in tubular structures or glands, mitotic rate and the degree of nuclear pleomorphism [10]. The grade of breast cancer is a reliable prognostic factor and is incorporated into decision making tools.

In multivariate models grade remains an independent prognostic factor for ER positive tumours [11]. Grade 1 and 3 breast carcinomas represent very different diseases, furthermore progression from high to low grade carcinoma from the study of molecular data is exceedingly rare [5].

The expression of ER, HER2 and PR status is assessed routinely in all invasive breast carcinomas. These markers are established predictive and prognostic factors. The expression of these markers is critical in guiding patient treatment in breast cancer patient’s [12].

ER receptor status is expressed in 80% of breast carcinomas and PR in 60–70 % [12,13]. ER positive tumours co-express PR in 70–80% of cases (ER+/PR+). The response to hormonal treatment varies, the best response is seen with ER+/PR + tumours and lower rates of response with ER+/PR- and ER-/PR + tumours. HER2 is expressed in 15–20% of primary breast carcinoma.^5^ HER2 positive breast carcinomas have the most unfavourable prognosis of all types of breast carcinoma. Breast carinomas that are “triple negative” do not express ER, PR or HER2. This is a extremely heterogenous group genetically, histologically, prognisically and in regards to treatment response [5].

Morphological intratumour heterogeneity can be appreciated as variability in different areas of tumour (spatial heterogeneity) or tumour progression over time (temporal heterogeneity) [14] Spatial heterogeneity can be appreciated within a single tumour or between primary breast carcinoma and synchronous lymph node metastases and even between synchronous metastases from different sites [5]. Breast carcinoma with truly mixed morphology consist of two morphologically different components for example IDC and mucinous carcinoma. Whereas other tumours exhibit ambiguous morphological features such as IDC with lobular features or contain foci of distinct differentiation for example IDC with spindle cell differentiation.

Temporal heterogeneity includes the evolution of invasive tumour in response to therapy or over time, and covers the progression from insitu to invasive carcinoma [[15], [16], [17], [18], [19], [20]].

Four mechanisms of breast cancer heterogeneity have been described which includes differentiation of state of the cell of origin, cell plasticity, genetic evolution of cancer and tumour microenvironment [5].

Receptor status change is dynamic and unstable throughout tumour progression and during advanced stage disease. Of the 20% of breast cancer cases that relapse one-third will have discordance between their original ER/PR status and relapsed status, and up to 15% have discordance of with HER2 status. Change in receptor status throughout tumour progression may have prognostic implications and treatment is changed in up to 14% of cases. Loss of receptor status in associated with decreased overall survical [21]. Using ER as a biomarker; 5–10% of multifocal cancers and approximately 20% of bilateral breast cancers are discordant.

ER status of metastases has been reported to differ from that of the primary cancer in 10–40 % of patients. [22]

Studies have found that cases with discordance of receptor status of metastatic disease and primary breast cancer have a worse prognosis than those who have ER positive concordance.

It is unclear whether heterogeneity of hormone receptor status predicts worse clinical outcomes among patients with synchronous bilateral breast cancer [[23], [24], [25]]. Heterogeneity in hormone-receptor status is of use in predicting the overall survival and breast cancer –specific survival. Furthermore, the prognostic value of ER status is of more utility than that of PR status.

There is a variation in the effect of the hormone-receptor status with respect to follow up time.

In patients with synchronous bilateral breast cancer ER discordance in patients have been associated with higher mortality than ER concordant positive patients and lower mortality than ER concordant negative patients within the first 5 years of surveillance. [25]

Studies support the need to evaluate hormone receptor status in all breast cancer lesions irrespective of tumour size or stage.

Heterogeneity in hormone receptor status alters the therapeutic management of patients with synchronous bilateral breast cancer. Both hormone therapy and chemotherapy should be considered in these patients.

## 4 Conclusion

Heterogeneity in hormone receptor status alters the therapeutic management of patients with synchronous bilateral breast cancer. Both hormone therapy and chemotherapy should be considered in these patients.

It is of utmost importance to evaluate the tumor receptor status in cases of synchronous bilateral breast tumour and to assess for change in relation to tumour progression or treatment. Further study in the status change of receptors could open up new treatment modalities.

There are no conflicts of interest or sources of funding.

## Conflict of interest

There are no conflicts of interest including employment, consultancies, stock ownership, honoraria, paid expert testimony, patent applications/registrations, grants or other funding.

## Funding source

There are no sources of funding for this research.

## Ethical approval

This study is exempt from ethical approval in this institution.

## Consent

Consent has been obtained from the patient.

## Author contribution

Dr Sunny Dhadlie - study concept - data collection, analysis, interpretation - writing the paper.

Contributers: Dr Rasika Hendahewa - study concept, Dr Joseph Whitfield - histopathology and images.

## Registration of research studies

Not applicable.

## Guarantor

Dr Rasika Hendahewa.

## Provenance and peer review

Not commissioned, externally peer reviewed.
